# Genetic Variants in DNA Mismatch Repair Pathway predict prognosis of Lung Cancer patients with receiving Platinum-Based Chemotherapy

**DOI:** 10.7150/jca.46150

**Published:** 2020-07-09

**Authors:** Jun-Yan Liu, Ting Zou, Ji-Ye Yin, Zhan Wang, Ying Wang, Zhao-Qian Liu, Juan Chen, Zhi-Wei Chen

**Affiliations:** 1 Department of Orthopaedics, The First Affiliated Hospital of the University of South China, Hengyang 421001, China.; 2 National Institution of Drug Clinical Trial, Xiangya Hospital, Central South University, Changsha, Hunan, P.R.China.; 3 Departments of Clinical Pharmacology, Xinagya Hospital, Central South University, Changsha 410008, China.; 4 Institute of Clinical Pharmacology and Hunan Key Laboratory of Pharmacogenetics, Central South University, Changsha 410078, China.; 5 Department of Medical Oncology, Lung cancer and Gastrointestinal unit, Hunan Cancer Hospital, Affiliated Cancer Hospital of Xiangya School of Medicine, Changsha 410013, China.; 6 Hunan clinical research center in gynecologic cancer, Hunan Cancer Hospital, Affiliated Cancer Hospital of Xiangya School of Medicine, Changsha 410013, China.; 7 Department of Pharmacy, Xinagya Hospital, Central South University, Changsha 410008, China.

**Keywords:** lung cancer, platinum-based chemotherapy prognosis, genetic polymorphisms, *MSH2*, * SAPCD1*, DNA mismatch repair

## Abstract

**Objective:** To investigate the relationships between genetic variants in DNA mismatch repair pathway genes and the prognosis of platinum-based chemotherapy in lung cancer patients.

**Methods:** 346 lung cancer patients who received at least two cycles of platinum-based chemotherapy were recruited in this study. A total of 35 single nucleotide polymorphisms in 7 DNA mismatch repair genes were genotyped to investigate their associations with platinum-based chemotherapy prognosis.

**Result:** The results revealed that patients carried *MSH2* rs4608577 TT genotype had a significantly shorter progression free survival than patients with GG or GT genotypes (Additive model: *P*=0.003, OR =0.94, 95% CI =0.33-1.57). Patients with *SAPCD1* rs707937 TT genotype had a significantly longer overall survival than patients with GG or GT genotypes (Additive model: *P*=0.0003, OR=0.75, 95% CI =0.35-1.14). Eight SNPs and fourteen SNPs were related to progression free survival and overall survival in subgroup analyses, respectively.

**Conclusion**: Our findings suggest that the* MSH2* rs4608577 and* SAPCD1* rs707937 may be potential clinical biomarkers for predicting platinum-based chemotherapy prognosis in lung cancer patients.

## Introduction

Lung cancer is the main causes of cancer-related mortality in the worldwide 1. Two major pathologic types of lung cancer are non-small cell lung cancer (NSCLC) and small cell lung cancer (SCLC), and NSCLC counted for about 85% of all cases 2, 3. Many new therapeutic agents such as gefitinib and erlotinib for target therapy were used for NSCLC patients, but it based on the genomic mutations of the patients. Thus, platinum-based chemotherapy regimens were still widely used for treatment of NSCLC in clinic 4-7. However, the prognosis of the lung cancer patients is still dissatisfactory and obstacle the treatment 8, 9. The tumor microenvironment state and clinical stage are the traditional prognostic factors 10-13, and many factors including genetic polymorphism, histology type, clinical stage, gender, age, and so on have been reported to be associated with prognosis of platinum-based chemotherapy 14-20. It is important to discern some new biomarkers that can be used to predict the prognosis of platinum-based chemotherapy.

The DNA repair has four major repair pathways including base excision repair (BER), nucleotide excision repair (NER), double-strand break repair (DSBR) and DNA mismatch repair (MMR). DNA mismatch repair (MMR) is a very important repair system which actuates DNA damage response 21, 22. It can correct errors during DNA replication. MMR proteins have been collected together to form protein complexes that recognize and digest mismatch pieces of DNA and eventually fill in the mismatches 23-27. Informally,* MutLα* plays a key role in the DNA mismatch repair system, and the endonuclease activity is related to DNA mismatch repair 28. The complexes of *MutLα* and the* MUTSα* complex can guide an act on repairing the single-base and small-loop mismatches, and the *MutSβ* complex also guides the repair of small-to large-loop mismatches 29. Therefore, *MutSα* and *MutLα* are important proteins in the MMR system and are used to cooperate the mismatch detection and repair events. In previous study, we found that* MutSα* and *MutLα* genes were associated with the toxicity of platinum-based chemotherapeutic in lung cancer 30, 31.

Recent study showed that mutations in DNA repair genes are related to the prognosis of platinum-based chemotherapy in NSCLC patients 32, 33. MMR plays a key role in removing cisplatin-DNA adducts that the formation of platinum/DNA adducts blocks replication and transcription of DNA, and the mutations of MMR genes can decrease the response to platinum-based therapies and influence the prognosis through the highly conserved biological pathway 34-37. The genetic polymorphisms of DNA mismatch repair genes were useful clinical biomarkers to predict chemotherapy response and prognosis in lung cancer patients 38.

In the current study, we genotyped 35 single-nucleotide polymorphisms (SNPs) in 7 MMR genes (*MLH1, MSH2, MSH3, MSH4, MSH5, SAPCD1 and MSH6*) in lung cancer patients, and evaluate the relationships between these genetic polymorphisms and platinum-based chemotherapy prognosis.

## Materials and Methods

### Subjects and follow up

All patients were selected by the following inclusion criteria: (1) patients newly diagnosed with lung cancer by histological examination at the Affiliated Cancer Hospital or Xiangya Hospital of Central South University (Changsha, Hunan, China) between August 2009 and January 2013; (2) patients who received at least 2 cycles of platinum-based chemotherapy; (3) patients with no history of surgery before chemotherapy. All patients provided written informed consent before they participated in this study. The study performed in accordance with the principles of Declaration of Helsinki and was approved by the Ethics Committee of Xiangya School of Medicine, Central South University.

The terminate date for patient follow-up was July 15, 2019. Survival data were collected by telephone follow-up or residence registration. Overall survival (OS) time was defined as the period between the date diagnosed of lung cancer and the date of last follow-up or death. Progression-free survival (PFS) was calculated from the date diagnosed of lung cancer until the date of the first local recurrence or metastases in the last follow-up. Patients without progression were censored at the date of the last contact.

### SNP selection, DNA extraction and genotyping

All of the common genetic variants in *MLH1, MSH2, MSH3, MSH4, MSH5, SAPCD1 and MSH6* were selected by Haploview (Broad Institute, Cambridge, MA, USA) using pair-wise tagging with default settings (pairwise r^2^ threshold = 0.8). SNPs with a minor allele frequency (MAF) ≥ 5% in the Han Chinese population were selected. Finally, Thirty-five SNPs were genotyped in the patients (Table 1). In our previous studies, the SNPs were investigated to be associated with platinum-based chemotherapy toxicity 30.

ALL blood samples were collected in the morning and stored in EDTA tube. Genomic DNA was isolated using a Genomic DNA Purification Kit (Promega, Madison, WI, USA) and stored at -20°C before use. Genotyping was analyzed using a Sequenom Mass ARRAY Genotyping Platform (Sequenom, San Diego, CA, USA) through polymerase chain reaction (PCR) system.

### Statistical analysis

The covariates for logistic regression included sex, age, smoking status, histology and clinical stage. We applied three genetic models (dominant, additive, and recessive) to evaluate the associations between the single-nucleotide polymorphisms and prognosis of the patients. The cumulative PFS and OS were estimated by using the log-rank test or univariate COX regression. All analyses were performed using PLINK (version 1.9, http://pngu.mgh.harvard.edu/purcell/plink/) and SPSS 18.0 software (SPSS Inc, Chicago, IL, USA). Odds ratios (OR) and their 95% confidence intervals (95% CI) were used to assess the associations between gene polymorphisms and prognosis. *P*<0.05 was considered statistically significant.

## Results

### Demographic characteristics of patient characteristics

A total of 346 patients who received first-line platinum-based chemotherapy were collected for this study. As shown in Table 2, the median age was 52 years old (a range of 21 to 77 years old). Among them, 286 (82.7%) were males and 60 (17.3%) were females. There were 224 (64.7%) patients who had ever smoked, and the rest 122 (35.3%) patients never smoked. For histology, 234 (67.6%) patients were diagnosed with NSCLC, and 110 (28.9%) with NSCLC. 294 (85%) of the patients were in an advanced stage (stage III/IV/ED). The fundamental clinical information, outcomes of progression-free survival (PFS) and Overall survival (OS) of these lung cancer patients are summarized in Table 2.

### Association of the *MSH2* rs4608577 polymorphisms and PFS in lung cancer patients

**Figure 1:**
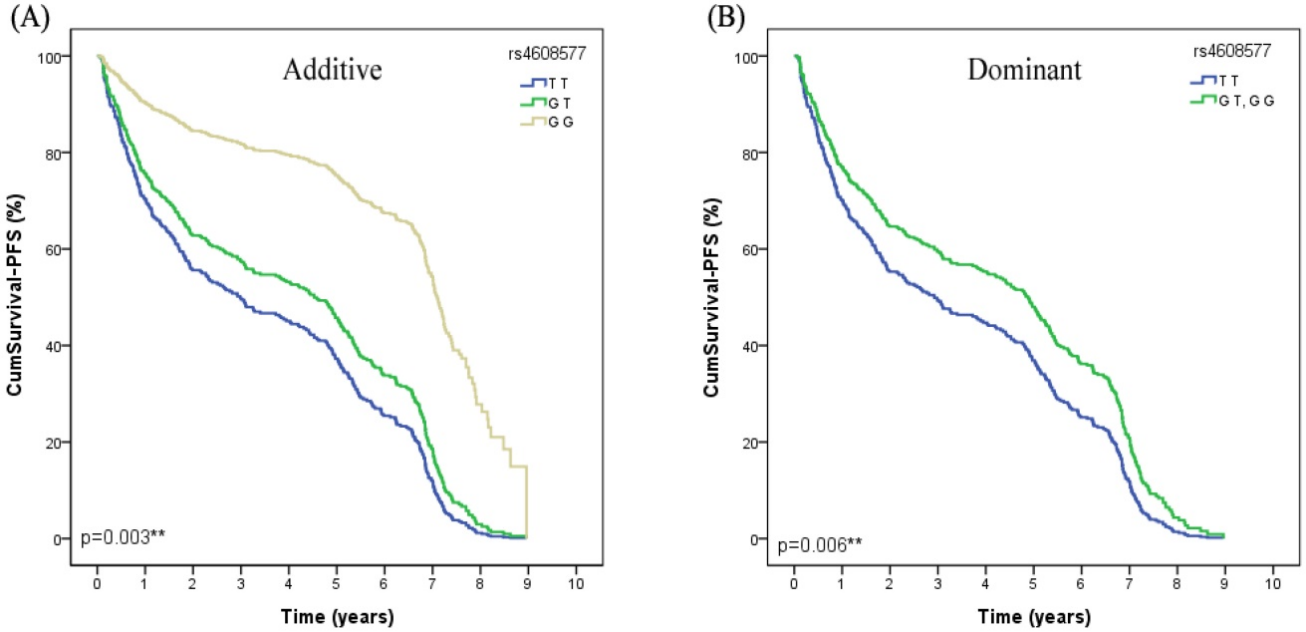
The MSH2 rs4608577 is significantly associcated with PFS in lung cancer patients treated with platinum-based chemotherapy. (**A**) Patients carry the genotype of MSH2 rs4608577 GG have a longer surival time than GT than TT in Additive model (*p*=0.003**). (**B**) Patients carry the genotype of MSH2 rs4608577 GG or GT have a longer surival time than TT in Dominant model (*p*=0.006**).

In the univariate Cox regression analysis, we found that *MSH2* rs4608577 was significantly associated with PFS of lung cancer patients received platinum-based chemotherapy. After adjusted by age, smoking status, gender, histological type and stage, rs4608577 still significantly contributed to PFS of lung cancer patients (Additive model: *P*=0.003, OR =0.94, 95% CI =0.33-1.57; Dominant model: *P*=0.006, OR=0.94, 95%CI=0.27-1.61) (Table 3). Patients carrying TT genotype had a significantly shorter PFS than patients carrying GG or GT genotypes (Figure 1).

### Association of the *SAPCD1* rs707937 polymorphisms and OS in lung cancer patients

**Figure 2:**
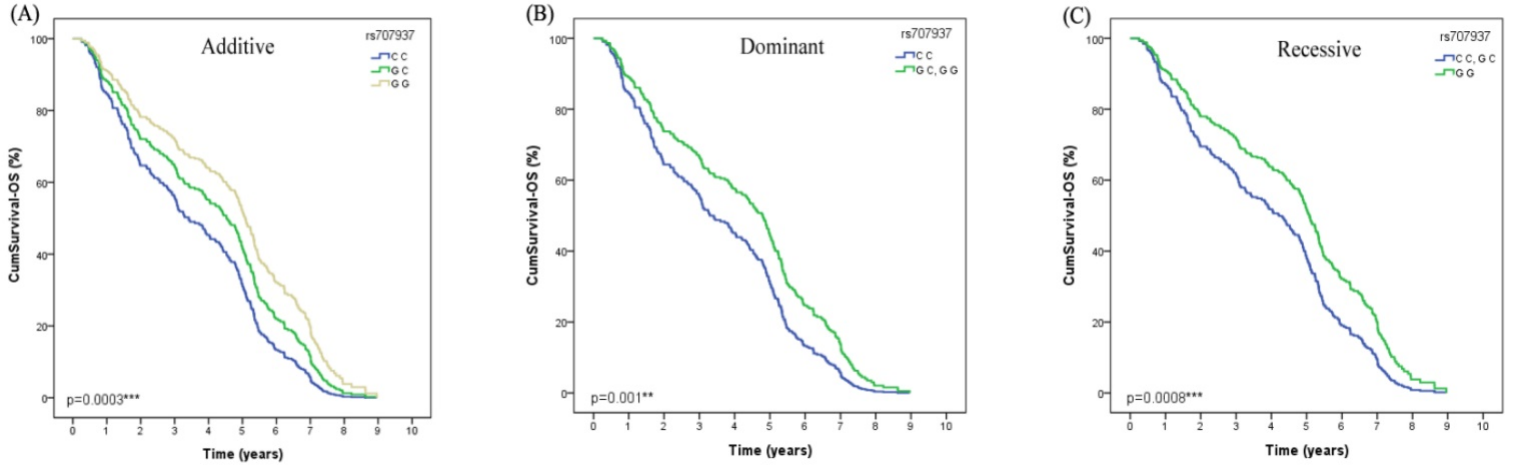
The polymorphism of SAPCD1 rs707937 is greatly related to OS in lung cancer patients of platinum-based chemotherapy treatment. (**A**) Patients with GG genotype have a longer overall survival time than GC than CC in Additive model (*p*=0.0003***). (**B**) Patients with GG or GC genotype have a longer overall survival time than CC in Dominant model in lung cancer patients (*p*=0.001**). (**C**) Patients with GG genotype have a longer overall survival time than GC or CC in Recessive model in lung cancer patients (*p*=0.0008***).

Both in the univariate Cox regression analysis and after adjusted by age, gender, smoking status, histological type and stage, we found that *SAPCD1* rs707937 was significantly associated with OS of lung cancer patients received platinum-based chemotherapy (Additive model: *P*=0.0003, OR=0.75, 95% CI =0.35-1.14; Dominant model: *P*=0.001, OR=0.97, 95% CI =0.38-1.56; recessive model: *P*=0.008, OR=0.98, 95% CI =0.26-1.70) (Table 4). In additive analysis model, patients who carried the rs707937 GG genotype had a significantly longer OS than patients with CC genotype. In dominant analysis model, patients who carried rs707937 GG and GC genotypes had a significantly longer OS than patients with CC genotype. In recessive analysis model, patients who carried rs707937 GG genotype had a significantly longer OS than patients with CC and GC genotypes (Figure 2).

### Stratification analyses

Stratification analyses were used to investigate the associations of all 35 SNPs with PFS and OS in the subgroups stratified by age, smoking status, gender, histological type and stage. For PFS subgroup analyses, we found the SNPs were related to PFS of lung cancer patients received platinum-based chemotherapy as follows:* MSH2* rs4608577 and *FBXO11* rs2710163 in patients with age >55 years old;* MSH3* rs6151914 in patients with age ≤55 years old;* MSH2* rs4608577 and *MSH6* rs3136329 in male patients; *CLIC1* rs2293852 in patients with smoking; *MSH2* rs4608577, *MSH3* rs6151914, *MSH4* rs3806162, *CLIC1* rs2293852 and rs805304 in NSCLC patients; *MSH3* rs1650665, rs26784 and rs6151670, and *MLH1* rs1800734 in SCLC patients; *MSH2* rs4608577, *MSH6* rs3136329 and *CLIC1* rs805304 in lung cancer patients with advanced or extensive stage (Table 5). For OS subgroup analyses, we found the SNPs were associated with OS of lung cancer patients received platinum-based chemotherapy in different subgroups as follows: *MSH2* rs2302428, *MSH3* rs6151914 and rs245346, *MSH4* rs5745532 and *SAPCD1* rs707937 in patients with ≤55 years old;* MSH3* rs245346 in patients with >55 years old; *MSH2* rs6544991, *MSH3* rs245346 and rs6151892 and *MSH6* rs2020910 in male patients; *MSH3* rs6151892 and rs3816729, and *MSH4* rs5745532 in patients with non-smoking; *MSH3* rs26778, *FBXO11* rs2710163 and *SAPCD1* rs707937 in NSLCL patients; *MSH2* rs2303428, *MSH3* rs6151914, *MSH5* rs707938 and rs31175772 in SCLC patients;* MSH3* rs6151914, *MSH4* rs3806162 and *FBXO11* rs3732190 in patients with early or limited stage; *MSH2* rs6544991 and rs13019654, and* SAPCD1* rs707939 in patients with advanced or extensive stage (Table 6).

## Discussion

In this study, we emphasize to investigate whether polymorphisms of 7 MMR genes (*MLH1, MSH2, MSH3, MSH4, MSH5, MSH6 and SAPCD1*) were related to the prognosis of platinum-based chemotherapy in 346 lung cancer patients. Our results showed that *MSH2* rs4608577 and *SAPCD1* rs707937 were significantly related to the prognosis of lung cancer patients received platinum-based chemotherapy. Patients with GG genotype of *MSH2* rs4608577 had better PFS, and patients with GG genotype of *SAPCD1* rs707937 would have better OS.

In the subgroup analyses for PFS, MSH2 rs4608577, MSH3 rs1650665, rs26784, rs6151670, rs6151914; *MSH4* rs3806162, *MSH6* rs3136329,* MLH1* rs1800734 were significantly associated with PFS in different subgroups. In the patients with older age (>55), we founded that the GG and GT genotype carriers in rs4608577 had significantly longer PFS when compared with TT genotype. For male patients, the GG and GT genotype also had significantly longer PFS. In adenocarcinoma subgroup, the hazard ratio for death was 1.29. Those patients with advanced stage carrying GG or GT genotype had longer PFS. For *MSH3* rs1650665, patients with squamous cell carcinoma carrying GA or GG had longer PFS. For *MSH3* rs26784, patients with squamous cell carcinoma carrying CC had longer PFS when compared with CT and TT. For *MSH3* rs6151670, patients with squamous cell carcinoma carrying GG or GC had shorter PFS when compared with CC. For *MSH3* rs6151914, patients with age ≤55 years old carrying CC or CT had longer PFS. Patients with adenocarcinoma subgroup carrying CC or CT had longer PFS. For* MSH4* rs3806162, Patients with adenocarcinoma subgroup carrying GG or GT had shorter PFS. For *MSH6* rs3136329, in male patients carrying CC or CT had longer PFS when compared with TT genotype. Patients with advanced or extensive stage carrying TT had longer PFS. For *MLH1* rs1800734, patients with squamous cell carcinoma carrying GG or GA had longer PFS.

In the subgroup analyses for OS, *MSH2* rs2303428, rs6544991, rs13019654 and *MSH3* rs6151914, rs245346, rs6151892, rs26778, rs3816729; *MSH4* rs5745532, rs3806162 and *MSH5* rs707938 rs3117572; *MSH6* rs2020910 and* SAPCD1* rs707937 were significantly associated with OS. We found the SNPs were related to OS of lung cancer patients received platinum-based chemotherapy as follows: *MSH2* rs2303428, patients with younger age (≤55) carrying TT or TC had longer OS when compared with CC. Patients with squamous cell carcinoma carrying TT or TC had longer OS. For rs6544991, in male patients carrying CC or CA had longer OS. Patients with advanced or extensive stage carrying CC or CA had shorter OS when compared with AA genotype. For rs13019654, patients with advanced or extensive stage carrying GG or GT had longer OS. For *MSH3* rs6151914, patients with younger age (≤55) carrying CC or CT had longer OS. Patients with squamous cell carcinoma carrying CC or CT had longer OS when compared with TT genotype. Patients with early or limited stage carrying CC had longer OS. For *MSH3* rs245346, patients with younger age (≤55) carrying TT or TC had shorter OS(recessive model OR=2.58, 95% CI=1.01,6.60, P=0.049) and older age (>55) carrying TT or TC had longer OS. In male patients carrying TT had longer OS. For rs6151892, in male patients carrying AA had longer OS when compared with AT or TT. Patients with non-smoking carrying AA or AT had longer OS. For rs26778, patients with adenocarcinoma subgroup carrying TT or TA had shorter OS when compared with AA genotype. For rs3816729, patients with non-smoking carrying CC or CT had longer OS. For *MSH4* rs5745532, patients with younger age (≤55) carrying TT or TC had longer OS and patients with non-smoking carrying TT or TC had longer OS. For rs3806162, patients with early or limited stage carrying GG or GT had longer OS. For *MSH5* rs707938, patients with squamous cell carcinoma carrying TT or TC had longer OS. For rs3117572, patients with squamous cell carcinoma carrying AA had longer OS when compared with AT or TT genotype. For *MSH6* rs2020910, in male patients carrying AA had longer OS when compared with AT or TT genotype. For *SPACD1* rs707937, patients with younger age (≤55) carrying GG or GC had longer OS. Patients with advanced or extensive stage carrying GG or GC had longer OS and also patients with adenocarcinoma subgroup carrying GG or GC had longer OS when compared with CC genotype.

In MMR system, the loss of functional MMR proteins such as *hMutSα* (*MSH2-MSH6*) and* hMutLα* (*MLH1-PMS2*) generate the tolerance with DNA alkylating agents. Apoptosis ensues directed by phosphorylation of p53 also related to these MMR repair gene functional in many cases 39. The functions of DNA mismatch repair act on DNA replication fidelity, mutation avoidance and genome stability, all of which contribute to the viability of organs and cells. The association of DNA mismatch repair gene polymorphisms and platinum-based chemotherapy toxicity in NSCLC patients have been reported 40, 41.

Gene variations in mutS homolog 2 (*MSH2*) have been founded to be related to tumor risk or survival in multiple kinds of tumors 42. Polymorphism in ASCL1 target gene DDC is associated with clinical outcomes of small cell lung cancer patients 43, 44. Nonetheless, there is no report about the association between OS or PFS and polymorphisms in the platinum resistance related *MSH2* and *SAPCD1* in Chinese patients. In our research, carrying GG or GT genotype in *MSH2* rs4608577 had better progression-free survival in lung cancer patients. Therefore, molecular study was needed to elucidate the mechanism of rs4608577 in* MSH2* that affects the MMR systems.

There are some limitations in this study. The samples were collected in the same region which also needed the multi-central study to overcome the analysis bias. Patients' sample size was not large enough to contribute to make results verification. The definite mechanism between *MSH2* rs4608577,* SAPCD1* rs707937 and the prognosis of platinum-based chemotherapy in lung cancer patients is still needed to be investigated. We hypothesized that rs4608577 and rs707937 were affect expression level of *MSH2* and *SAPCD1* respectively, and contributed to MMR activity.

In conclusion, our study showed that *MSH2* rs4608577 was significantly related to progression-free survival and *SAPCD1* rs707937 was significantly related to overall survival of platinum-based chemotherapy in lung cancer patients. *MSH2* rs4608577 and *SAPCD1* rs707937 perhaps could be biomarkers for predicting the prognosis in lung cancer patients with platinum-based chemotherapy.

## Figures and Tables

**Table 1:** The 35 single nucleotide polymorphisms examined in this study

Genes	SNPs	Alleles	Call rate (%)^#^	MAF^#^
MLH1	rs1800734	G/A	98.55	0.42
	rs1540354	A/T	97.98	0.32
MSH2	rs2303428	T/A, C, G	98.84	0.36
	rs10191478	G/T	98.27	0.19
	rs1981929	G/A,C	97.11	0.13
	rs4608577	T/G	97.40	0.13
	rs6544991	A/C	95.09	0.35
	rs7602094	T/C	95.09	0.32
	rs13019654	G/A, T	97.11	0.28
	rs12999145	A/G, T	99.13	0.46
	rs4952887	C/T	98.84	0.15
MSH3	rs6151627	A/G	99.13	0.26
	rs394592	G/A	99.13	0.49
	rs26784	A/G	98.27	0.37
	rs7709909	C/T	99.13	0.29
	rs1650665	C/T	99.71	0.04
	rs245340	G/A, T	97.69	0.27
	rs245346	A/G	98.27	0.42
	rs26778	A/T	96.53	0.42
	rs3816729	A/G	95.66	0.31
	rs6151670	C/G	98.55	0.26
	rs6151892	T/A	99.13	0.33
	rs6151914	C/T	98.27	0.10
MSH4	rs1146642	A/C, G	98.27	0.03
	rs3806162	T/G	99.13	0.23
	rs5745532	C/T	98.84	0.25
MSH5	rs707939	C/A	100	0.38
	rs1150793	A/G	99.42	0.04
	rs3117572	A/C, G	99.13	0.28
	rs707938	A/G	97.69	0.30
	rs409558	T/C	100	0.13
MSH6	rs2020910	T/A	98.55	0.18
	rs2348244	T/C	99.42	0.37
	rs3136329	C/T	97.98	0.13
SAPCD1	rs707937	C/G	96.53	0.43

MAF: Minor allele frequency; #Data in this study.

**Table 2:** Distribution of characteristics in lung cancer patients and prognosis analysis

Variables	Patients (N%)	Death (N%)	MST-OS (year)	MST-PFS (year)
**Age**				
≤55	154 (44.5)	127 (45.5)	4.52	3.09
>55	192 (55.5)	152 (54.5)	4.35	3.21
**Gender**				
Male	286 (82.7)	229 (82.1)	4.42	2.93
Female	60 (17.3)	50 (17.9)	4.77	4.47
**Histology**				
NSCLC	234 (67.6)	189 (67.7)	4.58	3.25
SCLC	100 (28.9)	80 (28.7)	4.35	3.10
**Smoking status**				
Non-smoker	122 (35.3)	97 (34.8)	4.83	4.60
Smoker	224 (64.7)	182 (65.2)	4.13	2.61
**Stage**				
I/II/LD	47 (13.6)	39 (14.0)	3.80	3.19
III/IV/ED	294 (85.0)	240 (86.0)	4.53	3.16

MST, median survival time; LD, Limitation Disease; ED, Extensive Disease.

**Table 3:** Association of the MSH2 rs4608577 polymorphisms and PFS in lung cancer patients

Gene	Polymorphism	Genotype	MPFS (year)	Additive		Dominant		Recessive	
				OR (95%CI)	*p* value	OR (95%CI)	*p* value	OR (95%CI)	*p* value
MSH2	rs4608577	GG	7.97	0.94 (0.33-1.57)	0.003**	0.94 (0.27-1.61)	0.006**	2.48 (-0.20-5.17)	0.071
		GT	4.94						
		TT	2.67						

MPFS, median progression-free survival; OR, odds ratio; CI, confidence interval; Additive model: comparison between minor allele subjects and major allele subjects. Dominant model: comparison between minor allele carriers and major homozygous subjects. Recessive model: comparison between major allele carriers and minor homozygous subjects. ** *P* < 0.01.

**Table 4:** Association of the SAPCD1 rs707937 polymorphisms and OS in lung cancer patients

Gene	Polymorphism	Genotype	MST (year)	Additive		Dominant		Recessive	
				OR (95%CI)	p value	OR (95%CI)	p value	OR (95%CI)	p value
SAPCD1	rs707937	GG	4.72	0.75 (0.35-1.14)	0.0003***	0.97 (0.38-1.56)	0.001**	0.98 (0.26-1.70)	0.008**
		GC	4.80						
		CC	4.05						

MST, median survival time; OR, odds ratio; CI, confidence interval; Additive model: comparison between minor allele subjects and major allele subjects. Dominant model: comparison between minor allele carriers and major homozygous subjects. Recessive model: comparison between major allele carriers and minor homozygous subjects. ** *P* < 0.01; *** *P* < 0.001.

**Table 5:** Stratification analyses of Association between the eight polymorphisms and PFS in lung cancer patients

Genes	SNPs	Subgroups	Additive		Dominant		Recessive	
			OR (95%CI)	*p* value	OR (95%CI)	*p* value	OR (95%CI)	*p* value
MSH2	rs4608577	Age (>55)	1.04 (0.22-1.86)	0.014*	1.10 (0.20-2.00)	0.017*	1.80 (-1.38-4.98)	0.268
		Male	0.86 (0.19-1.54)	0.013*	0.83 (0.09-1.53)	0.028*	2.61 (-0.08-5.31)	0.059
		NSCLC	1.29 (0.57-2.02)	0.001*	1.21 (0.42-2.00)	0.003*	4.45 (1.40-7.49)	0.005*
		Stage (III/IV/ED)	0.84 (0.18-1.51)	0.014*	0.81 (0.09-1.54)	0.029*	2.51 (-0.18-5.20)	0.068
MSH3	rs1650665	SCLC	1.82 (0.10-3.54)	0.041*	1.82 (0.10-3.54)	0.041*	Ref	
	rs26784	SCLC	-0.95 (-1.82- -0.08)	0.034*	-1.06 (-2.19-0.07)	0.068	-1.53 (-3.46-0.40)	0.123
	rs6151670	SCLC	-0.24 (-1.18-0.70)	0.619	0.17 (-0.94-1.29)	0.761	-2.65 (-5.14--0.16)	0.040*
	rs6151914	Age (≤55)	-1.20 (-2.19--0.21)	0.019*	-1.32 (-2.50--0.14)	0.030*	-2.48 (-5.55-0.59)	0.116
		NSCLC	-0.89 (-1.72--0.07)	0.034*	-0.96 (-1.87--0.04)	0.042*	-1.70 (-4.77-1.38)	0.280
MSH4	rs3806162	NSCLC	-0.05 (-0.61-0.51)	0.866	-0.32 (-0.97-0.33)	0.336	1.66 (0.01-3.31)	0.049*
MSH6	rs3136329	Male	0.72 (0.07-1.36)	0.030*	0.68 (-0.06-1.43)	0.074	2.10 (0.05-4.14)	0.045*
		Stage (II/IV/ED)	0.32 (-0.32-0.97)	0.326	0.16 (-0.59-0.91)	0.675	2.08 (0.04-4.13)	0.047*
MLH1	rs1800734	SCLC	0.91 (0.10-1.72)	0.029*	1.33 (0.17-2.50)	0.027*	0.95 (-0.58-2.48)	0.225

Additive model: comparison between minor allele subjects and major allele subjects. Dominant model: comparison between minor allele carriers and major homozygous subjects. Recessive model: comparison between major allele carriers and minor homozygous subjects. OR- odds ratio; CI- confidence interval; Ref.- reference. * *P* < 0.05.

**Table 6:** Stratification analyses of Association between the fourteen polymorphisms and OS in lung cancer patients

Genes	SNPs	Subgroups	Additive		Dominant		Recessive	
			OR (95%CI)	*p* value	OR (95%CI)	*p* value	OR (95%CI)	*p* value
MSH2	rs2303428	Age (≤55)	1.60 (0.95-2.70)	0.079	2.19 (1.04-4.61)	0.039*	1.33 (0.48-3.72)	0.583
		SCLC	1.71 (0.86-3.42)	0.127	3.33 (1.09-10.16)	0.034*	1.05 (0.31-3.56)	0.938
	rs6544991	Male	1.28 (0.86-1.89)	0.222	2.04 (1.16-3.59)	0.013*	0.62 (0.27-1.43)	0.266
		Stage (III/IV/ED)	0.90 (0.60-1.33)	0.586	1.24 (0.72-2.13)	0.434	0.34 (0.13-0.89)	0.027*
	rs13019654	Stage (III/IV/ED)	0.64 (0.42-0.98)	0.038*	0.58 (0.34-0.98)	0.041*	0.57 (0.21-1.53)	0.261
MSH3	rs6151914	Age (≤55)	2.90 (1.10-7.67)	0.032*	2.90 (1.05-7.99)	0.039*	Ref	
		SCLC	5.02 (1.58-15.93)	0.006*	5.21 (1.59-17.05)	0.006*	Ref	
		Stage (I/II/LD)	13.75 (1.39-136.4)	0.025*	13.75 (1.39-136.4)	0.025*	Ref	
	rs245346	Age (≤55)	1.34 (0.80-2.27)	0.269	1.00 (0.46-2.15)	1.000	2.58 (1.01-6.60)	0.049*
		Age (>55)	1.38 (0.88-2.16)	0.167	2.27 (1.10-4.67)	0.026*	0.94 (0.41.2.13)	0.880
		Male	1.50 (1.03-2.18)	0.036*	1.74 (0.97-3.14)	0.064	1.70 (0.88-3.26)	0.113
	rs6151892	Male	0.65 (0.43-0.99)	0.043*	0.61 (0.36-1.05)	0.073	0.49 (0.18-1.29)	0.148
		Non-Smoker	0.62 (0.33-1.16)	0.135	0.41 (0.18-0.95)	0.037*	1.13 (0.30-4.17)	0.860
	rs26778	NSCLC	1.21 (0.77-1.89)	0.410	0.89 (0.48-1.64)	0.706	2.66 (1.08-6.52)	0.033*
	rs3816729	Non-Smoker	0.55 (0.29-1.08)	0.082	0.39 (0.17-0.91)	0.030*	0.90 (0.23-3.59)	0.881
MSH4	rs5745532	Age (≤55)	2.16 (1.18-3.94)	0.012*	2.54 (1.22-5.27)	0.012*	2.61 (0.59-11.44)	0.204
		Non-Smoker	1.99 (1.05-3.77)	0.035*	2.86 (1.24-6.61)	0.014*	1.58 (0.40-6.29)	0.517
	rs3806162	Stage (I/II/LD)	0.10 (0.01-0.84)	0.034*	0.08 (0.01-0.76)	0.027*	Ref	
MSH5	rs707938	SCLC	0.41 (0.19-0.91)	0.029*	0.37 (0.14-0.94)	0.036*	0.25 (0.03-2.16)	0.207
	rs3117572	SCLC	2.09 (1.02-4.26)	0.043*	2.35 (0.92-6.02)	0.075	3.13 (0.69-14.20)	0.139
MSH6	rs2020910	Male	0.59 (0.35-0.98)	0.044*	0.61 (0.34-1.12)	0.110	0.16 (0.02-1.25)	0.080
SAPCD1	rs707937	Age (≤55)	1.90 (1.13-3.20)	0.016*	4.19 (1.67-10.55)	0.002*	1.38 (0.59-3.23)	0.455
		Stage (III/IV/ED)	1.54 (1.04-2.28)	0.030*	2.16 (1.18-3.96)	0.013*	1.36 (0.69-2.66)	0.373
		NSCLC	1.27 (0.83-1.95)	0.278	2.01 (1.00-4.02)	0.049*	0.89 (0.43-1.84)	0.748

Additive model: comparison between minor allele subjects and major allele subjects. Dominant model: comparison between minor allele carriers and major homozygous subjects. Recessive model: comparison between major allele carriers and minor homozygous subjects. OR- odds ratio; CI- confidence interval; Ref.- reference. * *P* < 0.05.
